# Letter to the Editor: Effect of pre-transplant viral therapy on post-transplant hepatitis B virus reactivation in patients with hepatitis B-related hepatocellular carcinoma

**DOI:** 10.1097/JS9.0000000000001553

**Published:** 2024-05-03

**Authors:** Shengke Zhang, Shangke Huang, Guanhu Yang, Hao Chi

**Affiliations:** a Department of Clinical Medicine, Southwest Medical University; b Department of Oncology, The Affiliated Hospital of Southwest Medical University, Luzhou, People’s Republic of China; c Department of Specialty Medicine, Ohio University, Athens, USA

*Dear Editor,*

Hepatitis B virus (HBV) infection is a critical determinant in the onset, progression, and recurrence of hepatocellular carcinoma (HCC), particularly in East Asia, where HBV prevalence is high. Timely intervention with anti-HBV therapy can substantially decrease both the incidence and recurrence of HBV-related HCC. Despite these measures, HCC continues to represent a significant global health burden, ranking as the sixth most prevalent cancer and the third leading cause of cancer death worldwide^1^. Current treatment protocols for HCC advocate liver transplant as the optimal therapeutic strategy for affected patients.

The retrospective cohort study by Huigang Li *et al*. published in the *International Journal of Surgery* ^2^, established that HBV reactivation post-transplant significantly impacts the prognosis of patients with HBV-related HCC. This analysis, conducted at two centers in China, involved patients before and after liver transplant, marking the first investigation into the correlation between post-transplant tumor recurrence, metastasis, and HBV reactivation. The study identified five predictors of overall survival (OS) post-transplant in HBV-related HCC patients: variables beyond the Milan criteria, pre-transplant AFP (alpha-fetoprotein) levels exceeding 400, poor tumor differentiation, microvascular invasion, and HBV reactivation. Additionally, factors such as being outside the Milan criteria, microvascular invasion, and HBsAg-positive grafts were independently associated with the risk of HBV reactivation. The constructed nomogram model provides significant predictive advantages for HBV reactivation, enhancing clinicians’ ability to foresee this condition and adjust post-transplant management strategies accordingly.

Despite its strengths, this study exhibits several limitations that may compromise the interpretation and generalization of the findings. The retrospective design and the study’s restriction to two centers could introduce biases associated with patient selection and regional treatment modalities, potentially limiting the applicability of the results to a broader, more diverse population. Furthermore, the absence of data on pre-transplant antiviral therapy may significantly influence the study’s outcomes. Huigang Li *et al*. demonstrated that some patients were unaware of their HBV infection prior to being diagnosed with HCC and did not receive adequate antiviral therapy before undergoing the transplant. This lack of uniform antiviral management pre-transplant could impact post-transplant prognosis. Patients informed about their HBV status early are typically placed on long-term antiviral therapy, which substantially lowers HBV DNA levels, with many achieving virological suppression – indicated by undetectable HBV DNA levels in the blood. Additionally, antiviral therapy can transition some patients from HBsAg-positive to negative, thereby reducing their viral load. In contrast, patients who received no or inadequate antiviral treatment may maintain high or fluctuating levels of HBV DNA, resulting in persistently high viral loads^3^. Consequently, disparities in antiviral therapy might change the composition of the study cohort and modify the risk of HBV reactivation among patients, thus confounding the findings to a certain extent.

Furthermore, while this study offers valuable insights into the association between HBV reactivation and HCC recurrence, particularly in the context of lung metastases, it falls short in examining the molecular characteristics of the tumor and the specifics of the immunosuppressive regimen applied post-transplant. Both factors are crucial in influencing reactivation rates and the progression of cancer^4^. A more comprehensive analysis that includes these dimensions would enhance our understanding of the interactions among viral biology, tumor genetics, and immune modulation, thereby providing a deeper insight into the mechanisms driving disease outcomes in this patient population.

In conclusion, the study by Huigang Li *et al*. offers a detailed analysis forecasting HBV reactivation in patients with HBV-related HCC undergoing liver transplant, utilizing various pre-transplant indices. While there is uncertainty regarding how much the extent of pre-transplant antiviral and post-transplant immunosuppressive therapies influence HBV reactivation, this does not undermine the overall conclusions of the study. This research significantly advances our understanding of HBV reactivation in the setting of liver transplant for HBV-related HCC. The utilization of multiple outcome metrics further underscores the efficacy of the proposed nomogram prediction model for assessing HBV reactivation risk. We commend the authors for their substantial contributions, which hold considerable value for clinical decision-making in this domain.

## Ethical approval

Not applicable.

## Consent

Not applicable.

## Sources of funding

This project is supported by the Doctoral Startup Fund of the Affiliated Hospital of Southwest Medical University (No. 19025) and the Innovation and Entrepreneurship Training Program of Southwest Medical University (No. 2023393).

## Author contribution

S.Z., S.H., G.Y., and H.C.: conceptualization, methodology, validation, formal analysis, investigation, resources, data curation, writing – original draft, writing – review and editing, supervision, and project administration.

## Conflicts of interest disclosure

There are no conflicts of interest.

## Research registration unique identifying number (UIN)

Not applicable.

## Guarantor

Shengke Zhang, Shangke Huang, Guanhu Yang, and Hao Chi.

## Data availability statement

Not applicable.

## Provenance and peer review

Not applicable.
